# Inflammation and Prolonged QT Time: Results from the Cardiovascular Disease, Living and Ageing in Halle (CARLA) Study

**DOI:** 10.1371/journal.pone.0095994

**Published:** 2014-04-25

**Authors:** Daniel Medenwald, Jan A. Kors, Harald Loppnow, Joachim Thiery, Alexander Kluttig, Sebastian Nuding, Daniel Tiller, Karin H. Greiser, Karl Werdan, Johannes Haerting

**Affiliations:** 1 Institute of Medical Epidemiology, Biostatistics and Informatics, Martin-Luther-University Halle-Wittenberg, Halle/Saale, Germany; 2 Department of Medical Informatics, Erasmus Medical Center Rotterdam, Rotterdam, The Netherlands; 3 Department of Medicine III, Martin-Luther-University Halle-Wittenberg, Halle/Saale, Germany; 4 Institute of Laboratory Medicine, Clinical Chemistry and Molecular Diagnostics, University of Leipzig, Leipzig, Germany; 5 German Cancer Research Centre, Division of Cancer Epidemiology, Heidelberg, Germany; University Heart Center Freiburg, Germany

## Abstract

**Background:**

Previous research found an association of CRP with QT time in population based samples. Even more, there is evidence of a substantial involvement of the tumor necrosis factor-alpha system in the pathophysiology of cardiac arrhythmia, while the role of Interleukin 6 remains inconclusive.

**Objective:**

To determine the association between inflammation with an abnormally prolonged QT-time (APQT) in men and women of the elderly general population.

**Methods:**

Data descend from the baseline examination of the prospective, population-based Cardiovascular Disease, Living and Ageing in Halle (CARLA) Study. After exclusion of subjects with atrial fibrillation and missing ECG recording the final study cohort consisted of 919 men and 797 women. Blood parameters of inflammation were the soluble TNF-Receptor 1 (sTNF-R1), the high-sensitive C-reactive protein (hsCRP), and Interleukin 6 (IL-6). In accordance with major cardiologic societies we defined an APQT above a QT time of 460 ms in women and 450 ms in men. Effect sizes and the corresponding 95% confidence intervals (CI) were estimated by performing multiple linear and logistic regression analyses including the analysis of sex differences by interaction terms.

**Results:**

After covariate adjustment we found an odds ratio (OR) of 1.89 (95% CI: 1.13, 3.17) per 1000 pg/mL increase of sTNF-R1 in women, and 0.74 (95% CI: 0.48, 1.15) in men. In the covariate adjusted linear regression sTNF-R1 was again positively associated with QT time in women (5.75 ms per 1000 pg/mL, 95% CI: 1.32, 10.18), but not in men. Taking possible confounders into account IL-6 and hsCRP were not significantly related to APQT in both sexes.

**Conclusion:**

Our findings from cross-sectional analyses give evidence for an involvement of TNF-alpha in the pathology of APQT in women.

## Introduction

A prolonged QT time is one of the most important electrocardiographic abnormalities, and an important cause of sudden cardiac death [1]. Because the QT time is generally longer in females, women are more often affected by arrhythmia due to a prolonged QT interval than men [2]. Over the last decades, research has mainly revealed insights in the genetic pathogenesis of a prolonged QT time [3]. However, the meaning of further pathophysiological mechanisms in the development of this abnormality is still the subject of research. In particular, the role of inflammation parameters and cytokines has rarely been examined. To our knowledge, there are only a few studies examining the association of inflammation and QT time [4], [5]. In a population-based sample, Kim et al. found a positive association of increased blood level of C-reactive protein (CRP) and length of the heart rate-corrected QT time (QTc). This is similar to the results in Kazumi et al. [4], who revealed again a significant association of CRP and QTc in a cohort of young healthy men. While the above-mentioned studies focused mainly on CRP, data examining additional inflammation parameters are still missing. Notably, the soluble tumor necrosis factor receptor 1 (sTNF-R1) might serve as a promising parameter as it was revealed that sTNF-R1 is a strong predictor for cardiovascular survival [6], [7]. In experimental studies, the TNF-alpha system – whose activity can be assessed by the plasma level of sTNF-R1 [8]– was found to influence calcium [9], [10] and potassium [11], [12] channels affecting QT time (shortening for the former and prolongation for the latter) and the susceptibility to arrhythmia [13]. The stability of sTNF-R1 makes it an easily assessable marker of the larger TNF system [14]. Furthermore, there is evidence from previous studies that interleukin 6 (IL-6) plays a major in role in the pathophysiology of cardiac arrhythmia [15], [16].

As prolonged QT time remains often undetected in apparently healthy subjects, it is typically a condition most relevant in (healthy) subjects of the general population, rather than patients in a clinical setting. Thus, the goal of the current study was to analyze the association between inflammation parameters, especially sTNF-R1, and prolonged QT time in the general population.

## Methods

### Study cohort

We used data from the ***CAR*** *dio-vascular Disease,* ***L*** *iving and* ***A*** *geing in Halle* study (CARLA study), which is a prospective population-based cohort study of the elderly general population of the city of Halle in eastern Germany [17], [18]. The CARLA cohort comprises 1,779 participants (baseline response 64.1%) aged 45–83 years at baseline (812 women, 967 men). The baseline examination took place between December 2002 and January 2006. A multi-step recruitment strategy aimed to achieve a high response rate. The percentage final response after subtracting exclusions (individuals who were deceased prior to the invitation, had moved away, or were unable to participate due to illness) was 64%. All data used in this cross sectional analysis descend from the baseline examination of the study. The study participants underwent a detailed medical examination and a standardized, computer-assisted interview, which collected information on socio-demographic and socioeconomic variables, behavioral, biomedical, and psychosocial factors, medical history, and the use of medication within the preceding 7 days. Medication was automatically coded according to the Anatomical Therapeutic Chemical Classification System (ATC code). Additionally, an analysis of non-respondents was performed in order to assess non-response bias by obtaining information about prevalent diseases, and selected behavioral and sociodemographic factors. A more comprehensive account of the CARLA study can be found in Greiser et al. [17]. The study was approved by the Ethics Committee of the Medical Faculty of the Martin-Luther-University Halle-Wittenberg and by the State Data Privacy Commissioner of Saxony-Anhalt and conformed to the principles outlined in the Declaration of Helsinki [19]. All participants gave written informed consent.

Subjects with electrocardiographic signs (n = 44) of atrial fibrillation assessed by Minnesota code [20] and a senior cardiologist were excluded.

### Laboratory measurements

Blood samples were taken after a supine rest of 30 minutes. The inflammation parameters of sTNF-R1 and IL-6 were analyzed by the Department of Medicine III, University Clinics Halle (Saale). After a 10-min centrifugation (20°C, 1,500 rpm, Acc = 9, Dcc = 3), the plasma was collected and stored at −80°C. The cytokines were determined using commercially available sandwich enzyme-linked immunosorbent assays (ELISAs: IL-6, Opteia, BD Biosciences, Heidelberg, Germany; TNF-R1, Boehringer Mannheim, Mannheim, Germany).

The determination of CRP was undertaken by the Institute of Laboratory Medicine, Clinical Chemistry and Molecular Diagnostics at the Leipzig University Clinics. The laboratory has been accredited according to the accreditation norms ISO 15180 and ISO 17025. Serum levels of high-sensitivity CRP (hsCRP) were measured using a high-sensitivity immunoturbidimetric method (CRP [Latex] HS, Roche, Mannheim, Germany) on a Hitachi autoanalyzer (Roche Diagnostics, Mannheim, Germany).

### Electrocardiogram (ECG) recoding

12-lead ECGs after a supine resting period of at least 20 min were recorded for 10 seconds (sec). All ECGs were processed by the Modular ECG Analysis System (MEANS) [21] to obtain the locations and types of the QRS complexes, and to assess the QT time in the 10-sec ECG. After detecting and, if possible, correcting artefacts by using an algorithm, the program takes all 12 leads into account and computes the QT time from a characteristic beat after considering further beats by an averaging process. Established as an objective method to assess peaks and intervals in ECGs previous studies ascertained the sufficient performance of the MEANS algorithm [22], [23]. In an independent test sample the algorithm identified all QRS (dominant type) complexes correctly (one false positive). Evaluating the accuracy of waveform recognition the difference in QT interval duration between MEANS and a reference standard was less than 2 ms with low variation [23].

We corrected the QT (QTc) time for heart rhythm by using the Bazett-Formula [24].

### Statistical analysis

According to the recommendations of major cardiologic scientific societies [25], we assumed an abnormally prolonged QT time (APQT) when the QTc was longer than 450 ms in men and longer than 460 ms in women [26]. In this article, we use the term ‘abnormally prolonged QT time’ rather than ‘long QT syndrome’, as the latter sometimes refers to underlying genetic abnormalities. Since the heart rate corrected QT time is computed using the uncorrected QT time and the recorded heart rate (HR) we included both parameters additionally in our analyses. All analyses were separately performed in men and women. Using logistic regression analyses with the binary outcome of existing APQT odds ratios with 95% confidence intervals (CI) were estimated as unadjusted and covariate adjusted values. Sex differences in the association of inflammation and QTc were assessed by incorporating an interaction term in the regression models. Non-linearity was assessed using restricted cubic splines which indicated that the assumption of linearity was a sufficient approximation of the exposure-outcome relation [27]. We used linear regression models (unadjusted and adjusted) in the analyses of QTc, QT and HR now as continuous variables. Respecting previous findings [5], [28], we adjusted our analyses for age, anti-arrhythmic (ATC code: C01B) and anti-phlogistic medication (ATC code: A07), current smoking status, high density lipoprotein (HDL), cholesterol, glucose blood level, alcohol intake, body mass index, thyroid stimulating hormone (TSH), systolic blood pressure, and potentially QT prolonging drugs (see www.qtdrugs.org) after reevaluating possible confounders by using directed acyclic graphs (DAG) [29]. With the assumed DAG model (see Figure S1) it is possible to estimate the total effect of inflammation on QTc. However, as mediation by electrolytes (not assessed in our study) is likely the direct effect cannot be estimated [30]–[32].

The adequacy of the considered regression models was assumed when the residuals were normally distributed, which was tested via a Q-Q plot and Cook's distance, which was required to be below one. In order to avoid possible confounding due to hormonal influences in females [33], we performed a sensitivity analysis excluding all premenopausal female subjects (n = 58, self-reported) and women with self-reported intake of sexual hormones (estrogens or gestagens) on a regular basis (n = 28) (detailed results are displayed in the supporting information). Additionally we performed a sensitivity analysis where all subject (120 men and 93 women) with regular intake of potentially QT prolonging drugs (see www.qtdrugs.org) were excluded from the analysis (detailed results are displayed in the supporting information).

The limit of statistical significance was assumed at an α of 5%. All statistical analyses and data management were performed using SAS, Version 9.3 (SAS Inc., Cary, NC, USA).

### Missing values

The following parameters incorporated in our analysis contained missing values (the number of missing values is given in brackets): IL-6 (121), hsCRP (82), sTNF-R1 (123), QTc (19), thyroid-stimulating hormone (21), glucose (12), cholesterol (12), HDL (12), triglycerides (12), current alcohol intake (3), and current smoking status (1). All other parameters were measured in all subjects. Using Student's t-Test and Chi-Square Test, we found no statistically significant differences when the mean values or frequencies, respectively, of the considered covariates (age, anti-arrhythmic [ATC code: C01B] and anti-phlogistic medication [ATC code: A07], current smoking status, HDL, cholesterol, glucose blood level, alcohol intake, and atrial fibrillation) were compared between subjects with missing inflammation parameters or QTc and subjects with complete data. As there were few missing values (maximum: 6.97% missing, sTNF-R1) and no evidence of bias or confounding due to incomplete data was evident, we conducted a complete case analysis.

## Results

### Baseline characteristics

Out of the 1,716 subjects with measured QT time, 123 (13.3%) men and 75 (9.4%) women showed a prolonged QTc according to our definition. Focusing on the inflammation parameters, plasma levels were homogenous between male and female subjects; only in the case of sTNF-R1 men showed higher plasma levels than women (see Table 1). Male subjects with APQT had only slightly higher blood values of sTNF-R1 than men without APQT. However, in female subjects with APQT, we found considerably higher sTNF-R1 plasma levels compared with women without such an electrocardiographic characteristic. The mean values of the inflammation parameter of IL-6 were lower in subjects without APQT then in subjects with APQT. In female and male subjects, hsCRP was higher in the APQT group than in the group with normal QTc (see Table 1). Out of the parameters taken as covariates into account, BMI appeared to be higher in the APQT subgroups of both sexes.

**Table 1:** Baseline characteristics of the CARLA collective – differentiated according to sex and presence of prolonged QT time.

	Men QTc < 450 ms	Men QTc > 450 ms	p†	Women QTc < 460 ms	Women QTc > 460 ms	p†
	[95% CI]	[95% CI]		[95% CI]	[95% CI]	
N*	796 (46,4%)	123 (7,2%)	-	722 (42.1%)	75 (4,4%)	-
cQT [ms]	417.79 [416.52, 419.07]	468.8 [465.85, 471.78]	-	423.11 [421.77, 424.46]	476.48 [473.03, 479.95]	-
sTNF-R1 [pg/mL]	1159.85 [1129.41, 1191.11]	1230.39 [1133.3, 1335.79]	0.1243	1047.56 [1019.81, 1076.05]	1266.6 [1148.62, 1396.69]	<.0001
IL-6 [pg/mL]	1.68 [1.55, 1.81]	2.26 [1.9, 2.69]	0.0045	1.83 [1.69, 1.97]	2.56 [1.95, 3.36]	0.0106
hsCRP [mg/L]	2.02 [1.86, 2.2]	2.61 [2.21, 3.08]	0.0251	1.85 [1.69, 2.02]	2.38 [1.86, 3.04]	0.0757
BMI [kg/m^2^]	27.65 [27.38, 27.92]	28.89 [28.13, 29.66]	0.0014	27.94 [27.57, 28.31]	29.73 [28.63, 30.88]	0.0047
Age [years]	62.87 [62.18, 63.58]	68.36 [66.66, 70.09]	<.0001	62.33 [61.63, 63.04]	66.88 [64.75, 69.09]	0.0002
Syst. BP [mmHg]	143.97 [142.65, 145.3]	150.13 [146.32, 154.05]	0.0013	139.79 [138.19, 141.4]	142.87 [138.53, 147.35]	0.2488
Glucose [mmol/l]	5.87 [5.78, 5.97]	6.33 [6.02, 6.67]	0.0008	5.62 [5.53, 5.7]	5.99 [5.66, 6.35]	0.0102
Cholesterol [mg/day]	5.29 [5.22, 5.36]	5.23 [5.05, 5.41]	0.531	5.63 [5.55, 5.71]	5.67 [5.4, 5.95]	0.7379
HDL [mmol/l]	1.23 [1.21, 1.25]	1.2 [1.15, 1.26]	0.3997	1.51 [1.48, 1.54]	1.51 [1.42, 1.61]	0.9586
Triglycerides[mmol/l]	1.77 [1.71, 1.85]	1.89 [1.72, 2.08]	0.2519	1.42 [1.37, 1.48]	1.48 [1.32, 1.66]	0.5051
TSH [mmol/l]	0.75 [0.71, 0.8]	0.82 [0.73, 0.92]	0.2876	0.75 [0.7, 0.81]	0.65 [0.49, 0.85]	0.1848
Alcohol [g/day]	1.48 [1.07, 2.05]	1.06 [0.44, 2.54]	0.4521	0.02 [0.01, 0.03]	0.04 [0.01, 0.12]	0.3864
	**Frequencies**					
Antiarrhythmic medication**	5 (0,6%)	1 (0,5%)	0.2187	4 (0,5%)	1 (1,3%)	0.3750
Antiphlogistic medication**	4 (0,5%)	1 (0,5)	0.3750	7 (1%)	1 (1,3%)	0.0703
QT prolonging medication **	89 (11.2%)	25 (20.3%)	0.0042	82 (11.4%)	10 (13.3)	0.6103

Geometric means with respective 95% confidence intervals.

Abbreviation: cQT: Bazett corrected QT interval; sTNF-R1: Soluble tumor necrosis factor type 1; hsCRP: High-sensitive C-reactive protein; IL-6: Interleukin 6; BMI: Body mass index; BP = Blood pressure; HDL: High density lipoprotein; TSH: Thyroid stimulating hormone.

* Proportion referred to the whole sample, ** Proportion within subgroup; †p-values refer to subgroups differences of subject with and without prolonged QT time within sexes.

In men, 25 (20.3%) of the subjects with a APQT received potentially QT prolonging drugs (Table 1), while the proportion was distinctly lower in women (13.3%).

### Cross-sectional association analysis

Using logistic regression models, sTNF-R1, but not hsCRP and IL-6, had a considerable association with APQT in female subjects. After adjustment for the considered covariates the odds ratio (OR) in women was 1.89 (95% CI: 1.13–3.17) per 1,000 pg/mL increase in sTNF-R1. In contrast, male subjects showed a much lower estimate (0.74, 95% CI: 0.48–1.15), which was accompanied by a considerable amount of uncertainty and was not statistically significant. The interaction analysis supported our finding of a circa 2.5-fold higher OR in women compared with men (OR: 2.55, 95% CI: 1.30–5.02), suggesting a significantly greater chance of APQT with increasing sTNF-R1 in women than in men.

**Figure 1:**
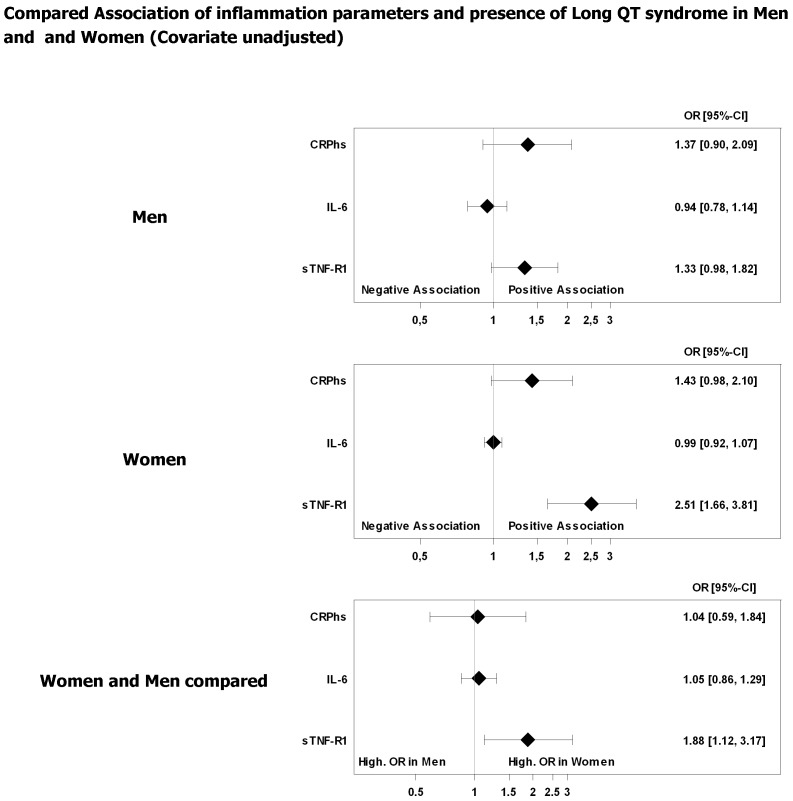
Association of prolonged QTc time and inflammation parameters. Odds ratios (OR) with 95% confidence interval. Unadjusted regression models of inflammation parameter. OR refers to a 1,000 pg/mL increase in sTNF-R1, a 10 pg/mL increase in IL-6, and a 10 mg/L increase in hsCRP.

**Figure 2:**
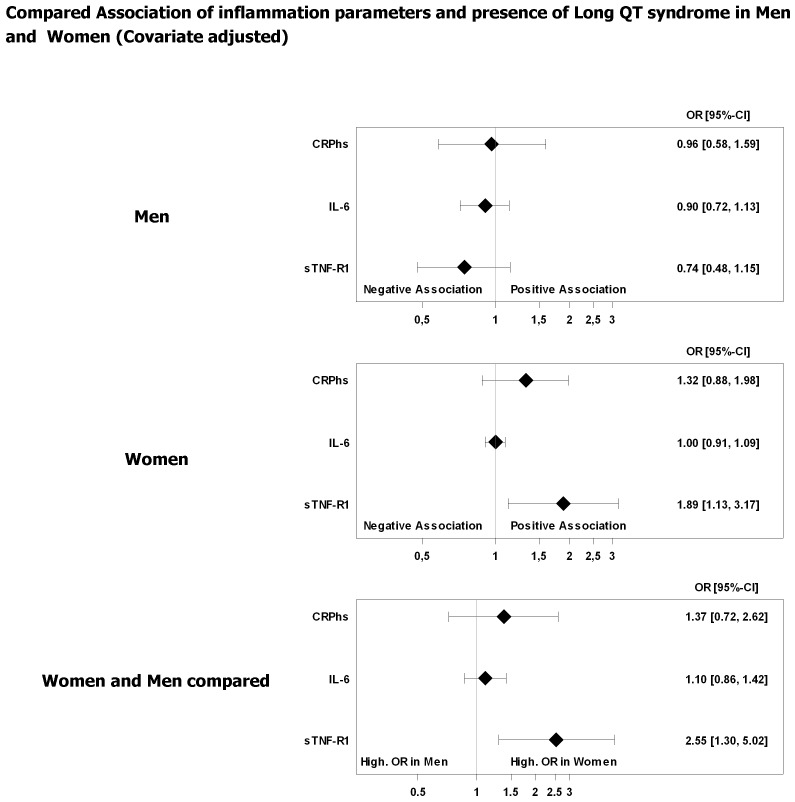
Association of prolonged QTc time and inflammation parameters. Odds ratios (OR) with 95% confidence interval. Models were adjusted for age, anti-arrhythmic (ATC code: C01B) and anti-phlogistic medication (ATC code: A07), current smoking status, high density lipoprotein (HDL), cholesterol, glucose blood level, alcohol intake, body mass index, thyroid stimulating hormone (TSH), systolic blood pressure and potentially QT prolonging drugs (see www.qtdrugs.org). OR refers to a 1,000 pg/mL increase in sTNF-R1, a 10 pg/mL increase in IL-6, and a 10 mg/L increase in hsCRP.

The estimated ORs of hsCRP and IL-6 appeared to reflect null effects, which is contrary to sTNF-R1 in female subjects (Figures 1, 2). Additionally, the results indicated no relevant sex-dependent association of hsCRP or IL-6 and APQT (Figures 1, 2).

**Figure 3:**
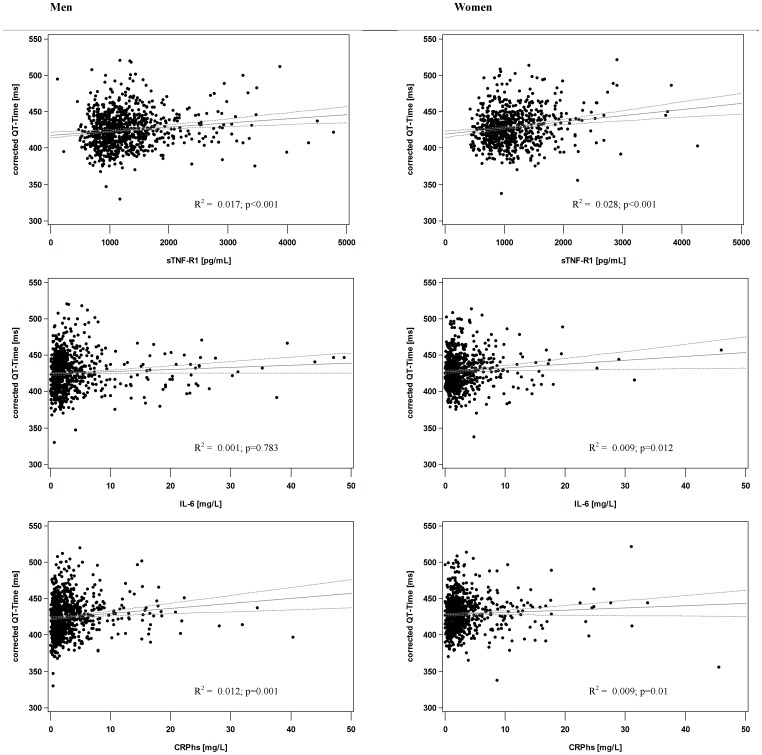
Scatter plot of corrected QT time dependent on sTNF-R1. x-axis: Soluble tumor necrosis factor type 1 (sTNF-R1) in pg/mL; y-axis: heart rate corrected QT time (QTc) in ms.

Analyzing the QTc as a continuous metric variable in a linear regression model adjusted for possible confounders we found again an association of sTNF-R1 and QTc in women (5.75 ms/1000 pg/mL, 95% CI: 1.32–10.18) but not in men (Table 2, Figure 3). The estimated association of QTc and hsCRP in the multiple analysis of both sexes was only minimal with wide confidence intervals (2.17 ms/10 mg/L, 95% CI: —1.96–6.3 in men; 2.02 ms/10 mg/L, 95% CI: −0.23–4.27 in women). In contrast to sTNF-R1, the inflammation parameter of IL-6 was negatively associated with QTc in women independently from possible confounders (0.51 ms/10 pg/mL, 95% CI: 0.09–0.93). In the separate analysis of HR and uncorrected QT time we found a positive association of hsCRP with HR (3.24 s^−1^/10 mg/L, 95% CI: 1.28–5.2), and consequently a negative relation with the uncorrected QT time in men. Interestingly, neither sTNF-R1 nor IL-6 was associated with either HR or uncorrected QT time in both sexes.

**Table 2:** Linear regression of QTc, QT time and heart rate (HR) on sTNF-R1 in men and women (estimates with 95% confidence interval).

QTc [ms]	Men* [95% CI]	Women*[95% CI]	Men**[95% CI]	Women**[95% CI]
sTNF-R1 [1000 pg/mL]	5.41 [2.52, 8.30]	8.46 [4.81, 12.11]	−0.22 [−3.44, 3]	5.75 [1.32, 10.18]
hsCRP [10 mg/L]	6.8 [2.68, 10.92]	2.94 [0.71, 5.18]	2.17 [−1.96, 6.3]	2.02 [−0.23, 4.27]
IL-6 [10 pg/mL]	−0.11 [−0.93, 0.7]	−0.54 [−0.96, −0.12]	−0.2 [−0.98, 0.59]	−0.51 [−0.93, −0.09]

QT [ms]	Men* [95% CI]	Women*[95% CI]	Men**[95% CI]	Women**[95% CI]
sTNF-R1 [1000 pg/mL]	6.13 [2.31, 9.95]	5.71 [0.88, 10.53]	1.54 [−2.73, 5.82]	2.97 [−2.86, 8.79]
hsCRP [10 mg/L]	−5.98 [−11.42, −0.54]	1.42 [−1.45, 4.28]	−8.36 [−13.85, −2.87]	1.14 [−1.77, 4.05]
IL-6 [10 pg/mL]	−1.04 [−2.11, 0.02]	−0.01 [−0.56, 0.54]	−0.95 [−1.99, 0.09]	0.01 [−0.54, 0.56]

Heart Rate [s-^1^]	Men* [95% CI]	Women*[95% CI]	Men**[95% CI]	Women**[95% CI]
sTNF-R1 [1000 pg/mL]	−1.36 [−2.75, 0.02]	0.21 [−1.41, 1.83]	−1.36 [−2.75, 0.02]	0.21 [−1.41, 1.83]
hsCRP [10 mg/L]	3.24 [1.28, 5.2]	0.43 [−0.51, 1.37]	3.24 [1.28, 5.2]	0.43 [−0.51, 1.37]
IL-6 [10 pg/mL]	0.39 [0.02, 0.76]	−0.11 [−0.29, 0.07]	0.39 [0.02, 0.76]	−0.11 [−0.29, 0.07]

Estimates refer to a 1,000 pg/mL increase in sTNF-R1, a 10 pg/mL increase in IL-6, and a 10 mg/L increase in hsCRP.

*unadjusted values; ** covariate adjusted values: models were adjusted age, anti-arrhythmic (ATC code: C01B) and anti-phlogistic medication (ATC code: A07), current smoking status, high density lipoprotein (HDL), cholesterol, glucose blood level, alcohol intake, body mass index, thyroid stimulating hormone (TSH), systolic blood pressure and potentially QT prolonging drugs (see www.qtdrugs.org).

OR refers to a 1,000 pg/mL increase in sTNF-R1, a 10 pg/mL increase in IL-6, and a 10 mg/L increase in hsCRP.

### Sensitivity analysis

The exclusion of premenopausal women and women taking oral hormones led to minor changes of the estimates, which were most relevant in the case of hsCRP where the covariate adjusted association with QTc increased and became statistically significant (see Table S1 and S2). Even more the negative association of IL-6 and QTc after covariate adjustment vanished, while the estimate in the covariate adjusted analysis of sTNF-R1 and QTc increased slightly.

After we excluded subjects with regular intake of potentially QT prolonging drugs effect estimates were not relevantly altered, while they increased slightly when the association of sTNF-R1 with QTc in women was considered (see Table S3 and S4).

## Discussion

Summarizing our results, sTNF-R1 seems to be more closely associated with APQT in women than in men, while there was no apparent association of hsCRP or IL-6 with QTc in both sexes. To our knowledge we are the first study that has shown the association of sTNF-R1 and prolonged QT time in women. Additionally, hsCRP was associated with heart rate and thus the uncorrected QT time rather than the actual heart rate corrected QT time. Certain parameters (such as BMI, age) were higher in subjects with APQT underlining their potential role as confounders in the case of a simultaneous effect on exposure and outcome. Considering them as covariates enables us to present results independently from a possibly confounding effect of these parameters (see also directed acyclic graphs in Figure S1). The decrease of effect sizes after covariate adjustment in our analyses might be in part explainable by the presence of confounding in univariate regression models.

Furthermore, we observed a high number of prevalent cases with APQT (11,6% of the entire cohort), which might be mainly due to an equally high prevalence of hypertension and thus ventricular hypertrophy in the CARLA cohort as it was shown previously [34]. Using a QTc cut off of 480 ms Johnson et al. found a prevalence of 13% in their collective of patients with hypertrophic cardiomyopathy [35].

Previous studies reported a considerable relationship between sTNF-R1 and cardiovascular diseases and, more specifically, survival in patients with symptoms of chronic heart failure [6]. It is well established that the TNF-alpha system is involved in the pathophysiology and progression of heart failure [36], [37]. Because the shedding of sTNF-R1 from the membrane-bound domain increases as the TNF-alpha system becomes more activated, sTNF-R1 might reflect the activity of TNF-alpha [8], [38]. A prolongation of the QT interval has its electrophysiological foundation in a prolonged action potential (AP) of myocardial cells; thus we would expect an increase in AP due to TNF-alpha. There is evidence from animal studies that TNF-alpha causes a decreased expression of various potassium (K^+^) channels [11] and a reduction in the density of cardiac transient outward K^+^ currents (I_to_) leading to an increase in AP duration. This mechanism was mediated by the inducible nitric oxide synthase (iNOS) [12], which was itself shown to be influenced by sex hormones (increased after ovariectomy [39]). Furthermore, ion channels (including potassium outward channels) of the human hearts have a different gene-distribution in men and women, which might also include a different susceptibility to TNF-alpha [40]. L-type Calcium channels are a further potential reason for the observed TNF-alpha relations. However, here the results are less conclusive: Recently, it was found that TNF-alpha was associated with a decrease in L-type Calcium currents, which would (contrary to our findings) result in a shortened QT interval [9], [10]. In contrast, Duncan et al. [13] reported a Calcium leakage caused by TNF-alpha provoking pro-arrhythmic events. A QT prolonging effect might be triggered, but was not examined by the authors. Summarizing previous experimental data K^+^ might play a key role in the observed relations; however these data refer mostly to animal studies rather than human beings. In line with our findings sex differences in the association of sTNF-R1 with cardiovascular events have been reported previously [41]. Interestingly, it was shown in experimental studies that testosterone leads to a decrease in TNF-alpha secretion in human macrophages. As we examined a collective of elderly subects it is unlikely that female hormones serve as an adverse contributor to the found relations in female subjects. Reflecting experimental data the withdrawal of estradiol was accompanied by an increase of TNF-alpha and iNOS [12] expression in ovariectomized rats [42]. This might imply that sex hormones in men and women have an inhibitory effect on the association of TNF-alpha and QT time. In line with this, the exclusion of premenopausal women or women with regular hormone intake caused a minor increase in estimates. However, this effect requires re-evaluation in a separate collective of younger (premenopausal) females to estimate the effect of sex hormones more reliable. In our cohort of the elderly population a direct influence of sex hormones on the estimated effects is less likely. Nevertheless, due to low case numbers in this subgroup we were not able to provide convincing evidence of hormonal effects.

The finding of a negative association of IL-6 with QTc is surprising as a positive association would be expected from previous studies [43]. However, the effect was of low magnitude and the statistical significance might be biased due to the low variance of this parameter in women. Significantly higher levels of CRP in AQPT subjects seem to be driven by the low variance of this parameter rather than a relevant association with QT time, which is underlined by the inconclusive results of regression analyses. A similar explanation might also be true for CRP underpinning that differences below 1 mg/L (and 1 pg/mL in the case of IL-6) are also not relevant from a practical point of view.

Apart from the correlation with heart rate, CRP seems to play a less important role in cardiac electrophysiology than the above-mentioned sTNF-R1. In one of the few studies having examined the association of inflammation and QT time effect sizes were small [5], which emphasizes the vague role of hsCRP in cardiac arrhythmia [44].

### Limitations

As we only used cross-sectional data, we failed to clearly determine the direction of the observed association, that is, whether inflammation caused a prolonged QT time or a long QT time led to higher blood levels of inflammation. At least for hsCRP, Kim et al. [5] have argued that an effect of prolonged QT time on inflammation is imaginable [45]. However, there is humble evidence that inflammation, besides TNF-alpha, influences cardiac ion channels [13], atrial fibrillation [15] and heart rate [46]. Future prospective studies with at least one follow-up might provide deeper insights in the causality of inflammation and prolonged QT time. The observational character of our study makes it difficult to provide deeper insights into underlying (patho)-physiologic mechanisms of our findings.

Furthermore, we only took three inflammation parameters into account. As sTNF-R1 might be implicated in other inflammatory mechanisms and related to a variety of other cytokines [47] further studies are needed that examine a larger variety of possible inflammation parameters. Due to the lack of genetic analyses we were not able to report the effect of inflammation in subjects with genetic anomalies related to a prolongation of QT time. Respecting the collective of the CARLA study our results apply mostly to the elderly population. Further studies with younger (female) subjects are warranted in order to clarify the influence of estrogens on QT time in more detail. Referring to our assumed DAG model (see Figure S1); we are not able to estimate the direct effect of inflammation on QT time (and vice versa) as electrolytes which possibly mediate the effect of inflammation on QT time were not measured in our study. However, it was our primary goal to estimate the total effect of this relation, which is possible with the used regression models [30]–[32].

In conclusion, our results underline the key role of the TNF-alpha system represented by its receptor in cardiac control circuits. Consequently, the effect of (anti-arrythmic) drugs on inflammation and TNF receptors might appear to be an interesting future research question [48], [49].

## Supporting Information

Figure S1 Directed acyclic graphs of parameters potentially influencing the association of inflammation and QT time. Minimal sufficient adjustment to estimate the total effect of inflammation of corrected QT time: age, blood pressure, blood fats/cholesterol, QT- prolonging drugs, smoking habit, and thyroid function. Minimal sufficient adjustment to estimate the direct effect of inflammation of corrected QT time: age, blood pressure, blood fats/cholesterol, QT- prolonging drugs, smoking habit, thyroid function, electrolytes.
(TIF)

Table S1 Linear regression of corrected QT time, QT time, and heart rate on inflammation parameters in women after exclusion of premenopausal women and women with regular hormone intake (estimates with 95% confidence interval). Estimates refer to a 1,000 pg/mL increase in sTNF-R1, a 10 pg/mL increase in IL-6, and a 10 mg/L increase in hsCRP. *unadjusted; ** covariate adjusted estimates: models were adjusted for age, anti-arrhythmic (ATC code: C01B) and anti-phlogistic medication (ATC code: A07), current smoking status, high density lipoprotein (HDL), cholesterol, glucose blood level, alcohol intake, body mass index, thyroid stimulating hormone (TSH), systolic blood pressure and potentially QT prolonging drugs (see www.qtdrugs.org).
(DOCX)

Table S2 Logistic regression of APQT on inflammation parameters in women after exclusion of premenopausal women and women with regular hormone intake (odds ratio with 95% confidence interval). Odds ratios refer to a 1,000 pg/mL increase in sTNF-R1, a 10 pg/mL increase in IL-6, and a 10 mg/L increase in hsCRP. *unadjusted Odds ratios; ** Odds ratios adjusted for age, anti-arrhythmic (ATC code: C01B) and anti-phlogistic medication (ATC code: A07), current smoking status, high density lipoprotein (HDL), cholesterol, glucose blood level, alcohol intake, body mass index, thyroid stimulating hormone (TSH), systolic blood pressure and potentially QT prolonging drugs (see www.qtdrugs.org). Abbreviation: APQT =  abnormally prolonged QT time.
(DOCX)

Table S3 Linear regression of corrected QT time, QT time, and heart rate on inflammation parameters in men and women after exclusion of subjects with regular intake of potentially QT prolonging drugs (estimates with 95% confidence interval). Estimates refer to a 1,000 pg/mL increase in sTNF-R1, a 10 pg/mL increase in IL-6, and a 10 mg/L increase in hsCRP. *unadjusted; ** covariate adjusted estimates: models were adjusted for age, anti-arrhythmic (ATC code: C01B) and anti-phlogistic medication (ATC code: A07), current smoking status, high density lipoprotein (HDL), cholesterol, glucose blood level, alcohol intake, body mass index, thyroid stimulating hormone (TSH), and systolic blood pressure.
(DOCX)

Table S4 Logistic regression of APQT on inflammation parameters in men and women after exclusion of subjects with regular intake of potentially QT prolonging drugs (odds ratio with 95% confidence interval). Odds ratios refer to a 1,000 pg/mL increase in sTNF-R1, a 10 pg/mL increase in IL-6, and a 10 mg/L increase in hsCRP. *unadjusted Odds ratios; ** Odds ratios adjusted for age, anti-arrhythmic (ATC code: C01B) and anti-phlogistic medication (ATC code: A07), current smoking status, high density lipoprotein (HDL), cholesterol, glucose blood level, alcohol intake, body mass index, thyroid stimulating hormone (TSH), and systolic blood pressure. Abbreviation: APQT =  abnormally prolonged QT time.
(DOCX)
