# Supplementary figures and images for: Inflammation and Prolonged QT Time: Results from the Cardiovascular Disease, Living and Ageing in Halle (CARLA) Study
Source: PLoS One. 2014 Apr 25;9(4):e95994. doi: 10.1371/journal.pone.0095994 (PMC4000193; doi:10.1371/journal.pone.0095994)

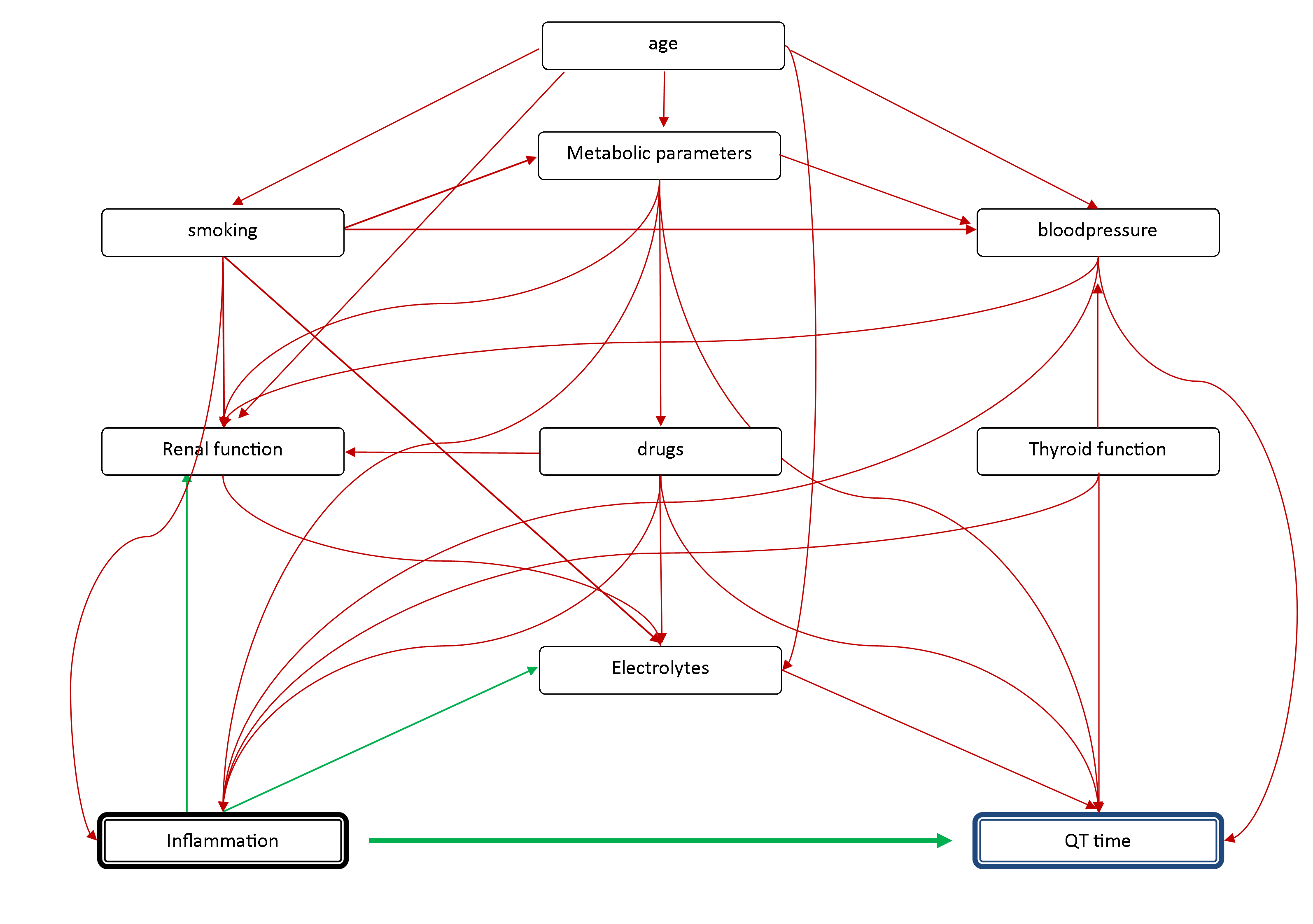

Supplement: Figure S1 — Directed acyclic graphs of parameters potentially influencing the association of inflammation and QT time. Minimal sufficient adjustment to estimate the total effect of inflammation of corrected QT time: age, blood pressure, blood fats/cholesterol, QT- prolonging drugs, smoking habit, and thyroid function. Minimal sufficient adjustment to estimate the direct effect of inflammation of corrected QT time: age, blood pressure, blood fats/cholesterol, QT- prolonging drugs, smoking habit, thyroid function, electrolytes. (TIF) [file pone.0095994.s001.tif]
