# Supplementary material for: Inflammation and Prolonged QT Time: Results from the Cardiovascular Disease, Living and Ageing in Halle (CARLA) Study
Source: PLoS One. 2014 Apr 25;9(4):e95994. doi: 10.1371/journal.pone.0095994 (PMC4000193; doi:10.1371/journal.pone.0095994)
Supplement: Table S1 — Linear regression of corrected QT time, QT time, and heart rate on inflammation parameters in women after exclusion of premenopausal women and women with regular hormone intake (estimates with 95% confidence interval). Estimates refer to a 1,000 pg/mL increase in sTNF-R1, a 10 pg/mL increase in IL-6, and a 10 mg/L increase in hsCRP. *unadjusted; ** covariate adjusted estimates: models were adjusted for age, anti-arrhythmic (ATC code: C01B) and anti-phlogistic medication (ATC code: A07), current smoking status, high density lipoprotein (HDL), cholesterol, glucose blood level, alcohol intake, body mass index, thyroid stimulating hormone (TSH), systolic blood pressure and potentially QT prolonging drugs (see www.qtdrugs.org). (DOCX) [file pone.0095994.s002.docx]

**Table S1: Linear regression of corrected QT time, QT time, and heart rate on inflammation parameters in women after exclusion of premenopausal women and women with regular hormone intake (estimates with 95% confidence interval).**

| **QTc [ms]** | Women*[95% CI] | Women**[95% CI] |
| --- | --- | --- |
| sTNF-R1 [1000 pg/mL] | 9.55 [5.63, 13.48] | 6.44 [1.69, 11.19] |
| hsCRP [10 mg/L] | 3.67 [1.35, 6] | 2.6 [0.24, 4.96] |
| IL-6 [10 pg/mL] | 0.82 [-0.56, 2.21] | 0.68 [-0.7, 2.06] |
| **QT [ms]** | Women*[95% CI] | Women**[95% CI] |
| sTNF-R1 [1000 pg/mL] | 5.94 [0.69, 11.2] | 4.54 [-1.75, 10.84] |
| hsCRP [10 mg/L] | 1.53 [-1.5, 4.56] | 1.18 [-1.91, 4.26] |
| IL-6 [10 pg/mL] | 0.05 [-1.78, 1.87] | -0.17 [-1.98, 1.65] |
| **Heart Rate [s-^1^]** | Women*[95% CI] | Women**[95% CI] |
| sTNF-R1 [1000 pg/mL] | 0.31 [-1.43, 2.05] | 0.57 [-1.48, 2.61] |
| hsCRP [10 mg/L] | 0.55 [-0.42, 1.53] | 0.41 [-0.58, 1.39] |
| IL-6 [10 pg/mL] | 0.1 [-0.49, 0.68] | 0.14 [-0.44, 0.72] |

Estimates refer to a 1,000 pg/mL increase in sTNF-R1, a 10 pg/mL increase in IL-6, and a 10 mg/L increase in hsCRP.

*unadjusted; ** covariate adjusted estimates: models were adjusted for age, anti-arrhythmic (ATC code: C01B) and anti-phlogistic medication (ATC code: A07), current smoking status, high density lipoprotein (HDL), cholesterol, glucose blood level, alcohol intake, body mass index, thyroid stimulating hormone (TSH), systolic blood pressure and potentially QT prolonging drugs (see [www.qtdrugs.org](http://www.qtdrugs.org)).
