# Supplementary material for: Inflammation and Prolonged QT Time: Results from the Cardiovascular Disease, Living and Ageing in Halle (CARLA) Study
Source: PLoS One. 2014 Apr 25;9(4):e95994. doi: 10.1371/journal.pone.0095994 (PMC4000193; doi:10.1371/journal.pone.0095994)
Supplement: Table S2 — Logistic regression of APQT on inflammation parameters in women after exclusion of premenopausal women and women with regular hormone intake (odds ratio with 95% confidence interval). Odds ratios refer to a 1,000 pg/mL increase in sTNF-R1, a 10 pg/mL increase in IL-6, and a 10 mg/L increase in hsCRP. *unadjusted Odds ratios; ** Odds ratios adjusted for age, anti-arrhythmic (ATC code: C01B) and anti-phlogistic medication (ATC code: A07), current smoking status, high density lipoprotein (HDL), cholesterol, glucose blood level, alcohol intake, body mass index, thyroid stimulating hormone (TSH), systolic blood pressure and potentially QT prolonging drugs (see www.qtdrugs.org). Abbreviation: APQT = abnormally prolonged QT time. (DOCX) [file pone.0095994.s003.docx]

**Table S2: Logistic regression of APQT on inflammation parameters in women after exclusion of premenopausal women and women with regular hormone intake (odds ratio with 95% confidence interval).**

| **APQT** | Women*[95% CI] | Women**[95% CI] |
| --- | --- | --- |
| sTNF-R1 [1000 pg/mL] | 2.71 [1.75, 4.20] | 2.02 [1.17, 3.47] |
| hsCRP [10 mg/L] | 1.50 [0.96, 2.34] | 1.29 [0.86, 1.95] |
| IL-6 [10 pg/mL] | 1.03 [0.89, 1.20] | 1.02 [0.87, 1.21] |

Odds ratios refer to a 1,000 pg/mL increase in sTNF-R1, a 10 pg/mL increase in IL-6, and a 10 mg/L increase in hsCRP.

*unadjusted Odds ratios; ** Odds ratios adjusted for age, anti-arrhythmic (ATC code: C01B) and anti-phlogistic medication (ATC code: A07), current smoking status, high density lipoprotein (HDL), cholesterol, glucose blood level, alcohol intake, body mass index, thyroid stimulating hormone (TSH), systolic blood pressure and potentially QT prolonging drugs (see [www.qtdrugs.org](http://www.qtdrugs.org)).

Abbreviation: APQT= abnormally prolonged QT time.
