# Supplementary material for: Inflammation and Prolonged QT Time: Results from the Cardiovascular Disease, Living and Ageing in Halle (CARLA) Study
Source: PLoS One. 2014 Apr 25;9(4):e95994. doi: 10.1371/journal.pone.0095994 (PMC4000193; doi:10.1371/journal.pone.0095994)
Supplement: Table S4 — Logistic regression of APQT on inflammation parameters in men and women after exclusion of subjects with regular intake of potentially QT prolonging drugs (odds ratio with 95% confidence interval). Odds ratios refer to a 1,000 pg/mL increase in sTNF-R1, a 10 pg/mL increase in IL-6, and a 10 mg/L increase in hsCRP. *unadjusted Odds ratios; ** Odds ratios adjusted for age, anti-arrhythmic (ATC code: C01B) and anti-phlogistic medication (ATC code: A07), current smoking status, high density lipoprotein (HDL), cholesterol, glucose blood level, alcohol intake, body mass index, thyroid stimulating hormone (TSH), and systolic blood pressure. Abbreviation: APQT = abnormally prolonged QT time. (DOCX) [file pone.0095994.s005.docx]

**Table S4: Logistic regression of APQT on inflammation parameters in men and women after exclusion of subjects with regular intake of potentially QT prolonging drugs (odds ratio with 95% confidence interval).**

| **APQT** | Men*[95% CI] | Men**[95% CI] | Women*[95% CI] | Women**[95% CI] |
| --- | --- | --- | --- | --- |
| sTNF-R1 [1000 pg/mL] | 1.19 [0.81, 1.74] | 0.64 [0.38, 1.08] | 2.82 [1.74, 4.58] | 2.30 [1.27, 4.18] |
| hsCRP [10 mg/L] | 1.31 [0.78, 2.20] | 0.94 [0.50, 1.77] | 1.41 [0.96, 2.07] | 1.32 [0.87, 2.00] |
| IL-6 [10 pg/mL] | 0.96 [0.79, 1.16] | 0.94 [0.73, 1.20] | 1.00 [0.93, 1.07] | 1.00 [0.92, 1.09] |

Odds ratios refer to a 1,000 pg/mL increase in sTNF-R1, a 10 pg/mL increase in IL-6, and a 10 mg/L increase in hsCRP.

*unadjusted Odds ratios; ** Odds ratios adjusted for age, anti-arrhythmic (ATC code: C01B) and anti-phlogistic medication (ATC code: A07), current smoking status, high density lipoprotein (HDL), cholesterol, glucose blood level, alcohol intake, body mass index, thyroid stimulating hormone (TSH), and systolic blood pressure.

Abbreviation: APQT= abnormally prolonged QT time.
